# Retrospective study on admission trends of Californian hummingbirds found in urban habitats (1991–2016)

**DOI:** 10.7717/peerj.11131

**Published:** 2021-04-13

**Authors:** Pranav S. Pandit, Ruta R. Bandivadekar, Christine K. Johnson, Nicole Mikoni, Michelle Mah, Guthrum Purdin, Elaine Ibarra, Duane Tom, Allison Daugherty, Max W. Lipman, Krystal Woo, Lisa A. Tell

**Affiliations:** 1 EpiCenter for Disease Dynamics, One Health Institute, School of Veterinary Medicine, University of California, Davis, Davis, CA, USA; 2 Department of Medicine and Epidemiology, School of Veterinary Medicine, University of California, Davis, Davis, CA, USA; 3 Department of Wildlife Fish and Conservation Biology, University of California, Davis, Davis, CA, USA; 4 Lindsay Wildlife Experience, Walnut Creek, CA, USA; 5 Santa Barbara Wildlife Care Network, Santa Barbara, CA, USA; 6 Wild Neighbors Database Project, Middletown, Middletown, CA, USA; 7 LA Wild, Los Angeles, CA, USA

**Keywords:** Anthropogenic threats, *Archilochus alexandri*, *Calypte anna*, *Calypte costa*, *Selasphorus rufus*, *Selasphorus sasin*, Wildlife rehabilitation, Wildlife rescue, California

## Abstract

**Background:**

Hummingbirds are frequently presented to California wildlife rehabilitation centers for medical care, accounting for approximately 5% of overall admissions. Age, sex, and reason for admission could impact hummingbird survivability, therefore identification of these factors could help maximize rehabilitation efforts.

**Methods:**

Mixed-effects logistic regression models were used to identify specific threats to the survival of 6908 hummingbirds (1645 nestlings and 5263 non-nestlings) consisting of five species (*Calypte anna, Calypte costa, Selasphorus rufus, Selasphorus sasin, Archilochus alexandri*), found in urban settings, and admitted to California wildlife rehabilitation centers over 26 years.

**Results:**

In total, 36% of birds survived and were transferred to flight cage facilities for further rehabilitation and/or release. Nestlings were more likely to be transferred and/or released compared to adult hummingbirds. After accounting for age, birds rescued in spring and summer were twice as likely to be released compared to birds rescued in the fall. A high number of nestlings were presented to the rehabilitation centers during spring, which coincides with the nesting season for hummingbirds in California, with the lowest number of nestlings presented in fall. Reasons for presentation to rehabilitation centers included several anthropogenic factors such as window collisions (9.6%) and interactions with domesticated animals (12.9%). Survival odds were lower if a hummingbird was rescued in a “torpor-like state” and were higher if rescued for “nest-related” reasons. Evaluation of treatment regimens administered at wildlife rehabilitation centers identified supportive care, including providing commercial nutrient-rich nectar plus solution, to significantly increase hummingbird survivability.

**Discussion:**

Our results provide evidence of threats to hummingbirds in urban habitats, based on reasons for rescue and presentation to rehabilitation centers. Reasons for hummingbird admissions to three California wildlife rehabilitation centers were anthropogenic in nature (i.e., being associated with domestic animals, window collisions, and found inside a man-made structure) and constituted 25% of total admissions. There was a clear indication that supportive care, such as feeding a commercial nectar solution, and medical treatment significantly increased the odds of survival for rescued hummingbirds.

## Introduction

Hummingbirds, found only in the Americas, are often presented to wildlife rehabilitation centers (Greenewalt, 1990; Heyden, 2005). Commonly found in urban settings due to the use of man-made feeders, hummingbirds are key pollinator species in urbanized areas (Maruyama et al., 2019). Hummingbird distribution (Greig, Wood & Bonter, 2017), population composition, and intra- and inter species interactions (Bandivadekar et al., 2018; Carpenter, 1987; Ditchkoff, Saalfeld & Gibson, 2006; Lowry, Lill & Wong, 2013; Ng et al., 2004; Thomas, Baker & Fellowes, 2014) all have been significantly affected due to increased artificial food-resource provisioning, and urbanization. Empirical studies describing disease and health risks in hummingbirds found in urban habitats are still needed.

Wildlife rehabilitation centers play a vital role in rehabilitation efforts and provide valuable data for wildlife commonly found in urban habitats (Griffith et al., 2013; Kelly & Bland, 2006; Molina-López & Darwich, 2011) through standardized medical records documenting success or failure for each wildlife rescue (Heyden, 2005; Kelly & Bland, 2006; Mazaris et al., 2008; Molina-López & Darwich, 2011; Wimberger & Downs, 2010). Evaluation of rehabilitation centers’ medical records can identify admission trends, key reasons for admission, anthropogenic threats (Deem, Terrell & Forrester, 1998; Griffith et al., 2013), and pathogen prevalences (Harris & Sleeman, 2007) for a wide variety of wildlife species. This vast availability of data brings a greater understanding of human-wildlife interactions in our urbanized world, as well as determines the overall impacts and outcomes of rescued wildlife following rehabilitation efforts (Molina-López & Darwich, 2011). With increasing numbers of birds being rescued daily and brought to rehabilitation centers (Deem, Terrell & Forrester, 1998; Molina-López & Darwich, 2011; Molina-López, Casal & Darwich, 2011), the analysis of data collected by rehabilitation centers provide a unique opportunity to understand and mitigate anthropogenic threats to hummingbirds.

To date and to the best of the authors’ knowledge, studies have not yet assessed outcomes for hummingbird rehabilitation. Hummingbird populations in urban settings are known to face a wide variety of anthropogenic threats, including domestic animal interaction, collisions with glass windows, and other manmade structures (Graham, 1997; Klem, 1989; Loss, Will & Marra, 2013), and destruction of nesting trees/shrubs. Circumstances that are the basis of hummingbirds’ presentation to rehabilitation centers could affect rehabilitation success and survival. Hence, evaluating anthropogenic threats and other factors such as species, age, and sex is critical for improving rehabilitation success. Furthermore, evaluating treatment options that increase the chances of successful rehabilitation will help prioritize efforts.

To address factors that impact the success of hummingbird rehabilitation, a retrospective study evaluating trends in presentation, treatment, and eventual disposition from data collected by three California wildlife rehabilitation centers was performed. Our objectives were to investigate the demographics of hummingbirds admitted to wildlife rehabilitation centers, to determine common presenting reasons for these hummingbirds being brought to the wildlife rehabilitation centers, to describe seasonal patterns of admission, and to examine whether age, sex, season, treatment administration, and/or reason for admission of a presented hummingbird influenced their rehabilitation outcome.

## Methods

**Figure 1:**
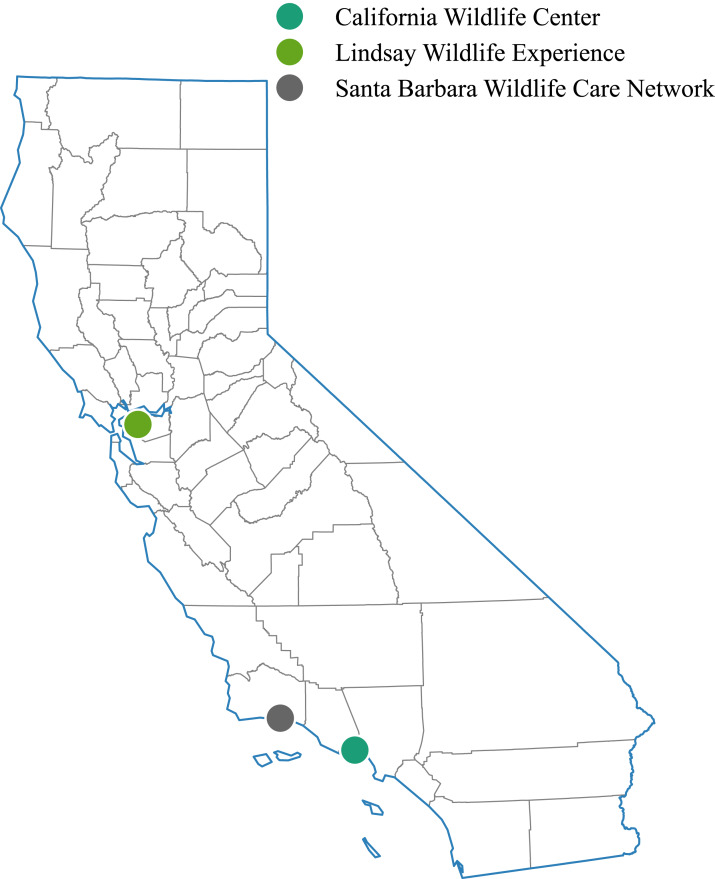
Wildlife rehabilitation centers contributing hummingbird rehabilitation data. Geographical map of the state of California highlighting the locations of the rehabilitation centers from where the hummingbird rehabilitation data records were obtained for this study (1991–2016).

Hummingbirds presented to wildlife rehabilitation centers were reported as nestling/adult on the ground unable to fly, sick, injured, or dead when they were brought into the wildlife rescue centers between January 1st, 1991 and December 31st, 2016. On admission, the rehabilitation center staff collected the following information for each bird: age, sex, species, the reason for admission, date of admission, and the date and place the bird was found. Veterinary technicians or veterinarians completed physical examinations and recorded administered treatments. All data were entered into an online database software called Wildlife Rehabilitation Medical Database (WRMD: https://www.wrmd.org/). Data used for the current study was imported from this database for the three rehabilitation centers. Admission records for hummingbirds presented to Lindsay Wildlife Experience (data for 1991–2016), Santa Barbara Wildlife Rescue Center (data for 2016), and California Wildlife Center, Malibu (data for 2013–2016), all located in California, were used for analysis. Figure 1 illustrates the locations of the rehabilitation centers.

### Standardization of data

#### Hummingbird identification

The WRMD data for hummingbird age were initially classified into two main categories: “nestling” and “adult-like bird” hummingbirds. All nestlings were considered unknown for sex and were classified into species groups (*Selasphorus* spp. and non-*Selasphorus* spp.) based on known breeding ranges and seasons of hummingbird species in California, and associated information reported by rescuers. The adult-like birds were further classified as “*Selasphorus* spp.” and “non-*Selasphorus* spp.” (Russell & Russell, 2001). Identification of adult males is less challenging for hummingbird species found in California due to their vibrant, distinct gorget colors compared to their counterpart adult females or young birds (Russell & Russell, 2001). For non-*Selasphorus* spp. (Anna’s Hummingbirds (ANHU), Black-Chinned Hummingbirds (BCHU) and Costa’s Hummingbirds (COHU)), records mentioning adult males were assigned “adult-like” and “male” for age and sex respectively. All other non-nestling and non-*Selasphorus* spp. hummingbirds were categorized as “unknown” and “female-like” for age and sex respectively. Similarly, for *Selasphorus* spp., records mentioning adult males (Allen’s Hummingbirds [ALHU] and Rufous Hummingbirds [RUHU]) were termed as “adult-like” and “male” for age and sex respectively. All other non-nestling *Selasphorus* spp. hummingbirds were considered “unknown” and “female-like” for age and sex. Lastly, records for hummingbirds without signalment information were categorized as “unknown” for age, sex, and species.

#### Reasons for admission

Data for reasons for admission were manually classified into seven categories depending on where and how a hummingbird was found by a good samaritan(s). Two categories were based on the location where the hummingbirds were found. Hummingbirds that were found on a patio, sidewalk, driveway, road, pool, or grass lawn were classified as “found on the ground” whereas hummingbirds that were found inside a human-built structure (e.g., a house, shop, garage, office building) were classified as “found inside”. If a hummingbird was found with a cat, dog, or in one case a chicken, the reason for admission was designated as “associated with domestic animal”. Hummingbirds that were associated with or found in the vicinity of a known nest were identified with the reason of “nest-related”. These included nestlings that were found fallen on the ground but the rescuer had been observing them on a nearby known nest, as well as nestlings that were rescued along with the nest, either where the nest was abandoned by the parents and was easy to extract from the tree/location or where the nesting trees/shrubs were cut down and the nest was found with the nestlings. Hummingbirds that were found at the feeder, usually unresponsive and sitting or hanging upside down, as well as hummingbirds sitting on a bush or fence that people were able to capture with minimal effort, were classified as “torpor-like state”. Another reason for admission included “window collisions” where hummingbirds were brought in after a collision with glass windows, windshields of parked vehicles, and glass doors. Lastly, entries where the reason for admission was not mentioned were classified as “unknown”.

#### Supportive measures and treatment provision

Based on the data available for supportive care and treatment provided, a binary variable (0 = treatment not provided, and 1 = treatment provided) was generated. Additional binary variables were created detailing the type of supportive care or treatment including (1) the provision of heat as a supportive measure for possible shock, (2) administration of oral fluids like commercial nectar plus solution as hydration and/or for energy supplementation, and (3) administration of medications like a non-steroidal anti-inflammatory (meloxicam; dosage 1.0 mg/kg on admission to the rehabilitation center and 0.5 mg/kg BID for 3–5 days) and steroids (dexamethasone) as well as antibiotic therapy (enrofloxacin; dosage 15 mg/kg BID 7–10 days). All treatments included in the study were provided by rehabilitation centers.

### Data analysis

Since none of the submitted birds had leg bands, all rescued hummingbirds were assumed to be first-time rescues for the purpose of this study. Two mixed-effects logistic regression models were developed to predict the final disposition of hummingbirds (survival or death) during the rehabilitation process. The first model looked at all the individuals while the second model was developed included only a subset of individuals who received preliminary treatment. Survival was defined when birds were transferred to flight cage facilities for further rehabilitation and/or released, or when nestlings were transferred to nurseries and no death or euthanasia was reported by rehabilitation centers. Species and sex groups were included as random effects. Model candidates were fitted and were compared with each other to identify best-fitting models based on AIC and ANOVA test. For the first model (model 1), factors related to demography and whether treatment was provided were tested, and reasons for admissions were explored. A second model predicting survival was developed that included only a subset of individuals whose records indicated that they received preliminary treatment at rehabilitation centers (model 2). Binary variables for each treatment option (heat, nectar/oral fluids, steroid, NSAID, antibiotic) were generated and included in the model. We assumed that reason for admission also accounted for the physical condition of the bird at the time of admission, which may have significantly affected the treatment options. The models were developed in R using the “glmmTMB” package (Brooks et al., 2017). For both models, an interaction term between age and seasons was included and resulting models were tested against the baseline model using the ANOVA test. Ten thousand simulations, only of the best fitting model 2, were used to predict the probability of survival for all the birds and outcomes were plotted against risk factors categories. The data used for the study, python and R code used for pre-processing the data, creating models and generating figures are openly stored in a Zenodo repository https://zenodo.org/record/4311820.

## Results

### Descriptive statistics of admission records

**Figure 2:**
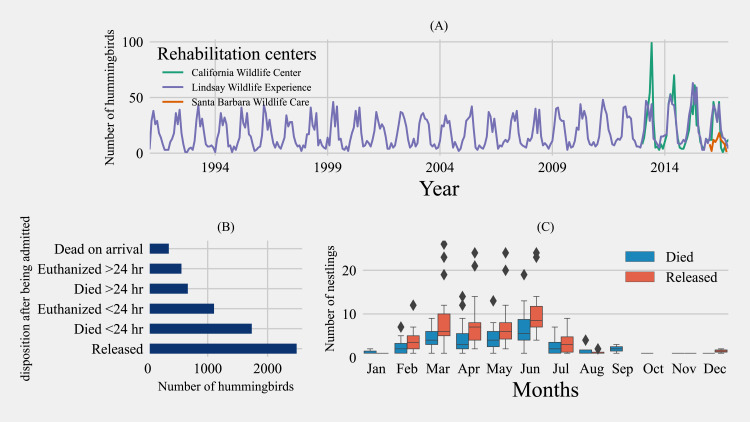
Hummingbird rehabilitation from 1991 to 2016 in Californian rehabilitation centers. (A) Temporal distribution of the number of birds admitted to California wildlife rehabilitation centers from 1991 to 2016. (B) Overall disposition distribution for rescued hummingbirds over a period of 26 years. (C) Distribution of nestling disposition by month rescued over 26 years (1991–2016).

A total of 6908 hummingbirds rescued from 192 city/town areas were presented to the three Californian rehabilitation centers involved in this study over 26 years. There was a distinct trend in the yearly distribution of rescued hummingbirds, with summer, followed by spring, being the most common rescue season and the winter season being the least common (Fig. 2A). Of the total birds rescued, 36.0% (*n* = 2485) were transferred to the flight cage to be returned to a free-ranging environment while the rest included birds that were dead on arrival (5.1%, *n* = 351), died during the process of rehabilitation (34.8%, *n* = 2404), and birds that needed to be euthanized during the process of rehabilitation (24.4%, *n* = 1668) (Fig. 2B). A total of 5723 non-*Selasphorus* spp. and 1,185 *Selasphorus* spp. hummingbirds were rescued. Out of 1645 nestlings, 35.7% (*n* = 587) either died or were euthanized, with higher nestling deaths reported between March and June (Fig. 2C). Similarly, within 5263 adult birds, 72.9% either died or were euthanized.

**Figure 3:**
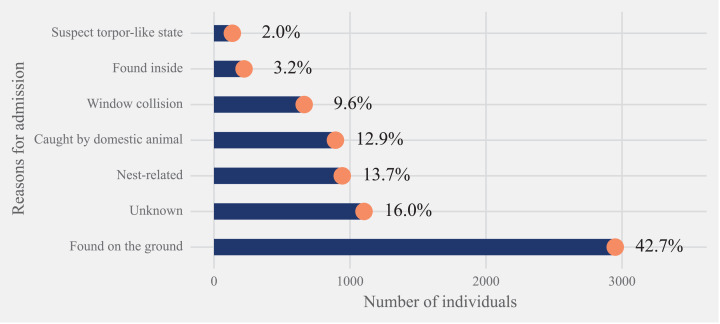
Reasons for hummingbird admission. Distribution of the rescued 6908 hummingbirds from the three Californian-based wildlife rehabilitation centers based on the reason for admission category.

**Figure 4:**
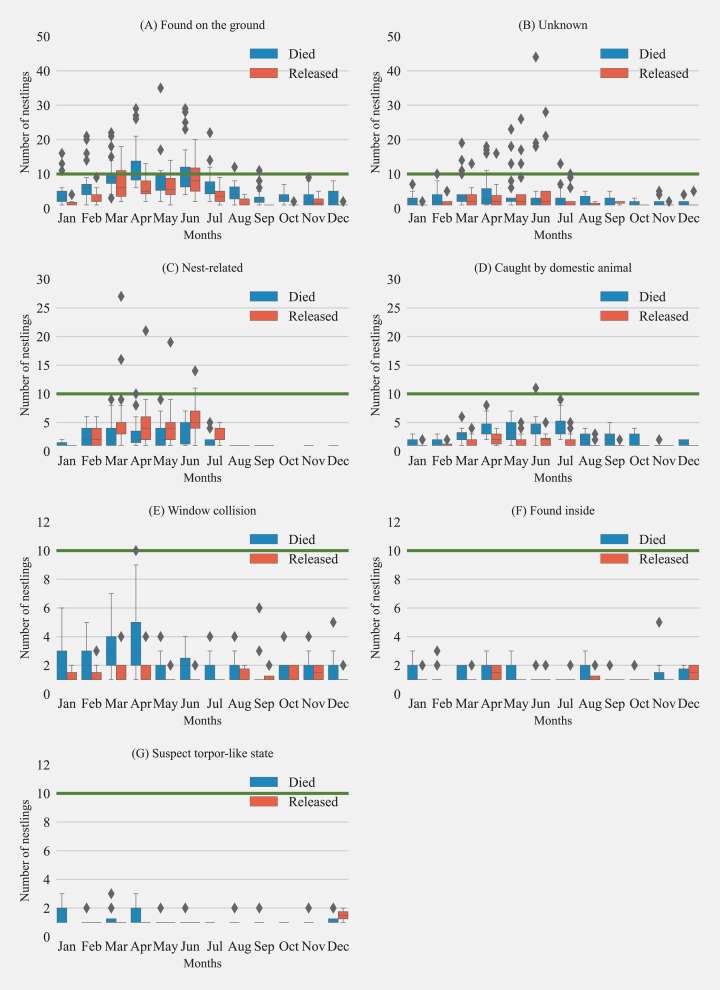
Monthly distribution of hummingbirds admitted to California wildlife rehabilitation centers and their disposition for each admission category (*n* = 7). (A) Found on the ground, (B) unknown, (C) nest-related, (D) caught by domestic animal, (E) window collision, (F) found inside and (G) suspect torpor-like state. Boxplots show the median, first, and third quartiles of the data. Whiskers extend to the data range and outliers are presented as separate diamonds. Green horizontal line represents 10 monthly cases for visual comparisons across subplots.

For the study period, the most common reason for admission was ‘found on the ground’ at 42.7% (*n* = 2950) and at 2.0% (*n* = 135) the least common was “torpor-like state” (Fig. 3). Of the total cases, 13.7% of rescue were “nest-related”. This was followed by 12.9% of hummingbirds that were admitted after being caught by a domestic animal, with most of them being associated with cats except for three instances (2 dogs and 1 companion chicken). Finally, 9.6% of cases were associated with “window collisions” (Fig. 3). The number of hummingbirds rescued after being “found on the ground” and “caught by domestic animals” showed seasonality components with a higher number of individuals with these reasons for rescue found in early spring to summer seasons. No birds were rescued for “nest-related” reasons between August to December (Fig. 4).

A model with age, season, treatment, and reasons for admission as fixed effects was identified as the best fitting model (AIC = 8137.40). Inclusion of an interaction term between age and season significantly improved the model fit (ANOVA Chisq *p* < 0.001, Table S1) and resulted in estimates for interactions that were statistically significant. Hence an interaction term for season and age was considered in the final model (model 1, AIC = 8131.2, *n* = 6908, Table S2). For the model evaluating treatment options (model 2), the inclusion of the interaction term between season and age did not improve the model fit (ANOVA Chisq *p* = 0.15, Table S3), hence no interaction term was included. The subset model evaluating treatment options (model 2) included binary variables for treatment options for heat, nectar/oral fluids, steroid, NSAID, and antibiotic administration along with demographic factors included in the general model (*n* = 3779, AIC = 4649.6, Table S4).

#### Effects of age and season on the release

**Figure 5:**
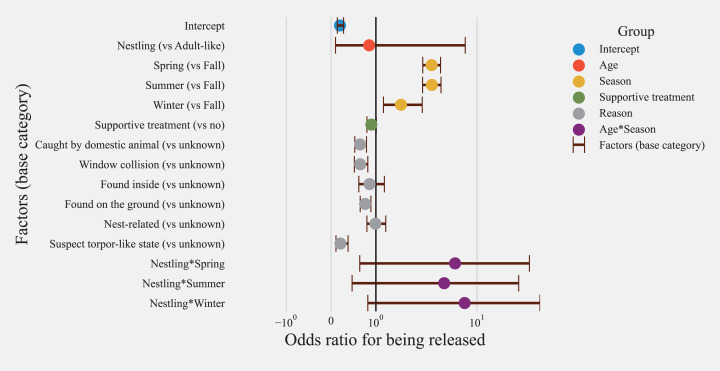
Risk factors affecting release of hummingbirds from rehabilitation centers. Odds ratios for all risk factors and their confidence intervals for rescued hummingbirds (*n* = 6908) with available treatment information. Categories are color-coded according to the group of independent variables.

**Figure 6:**
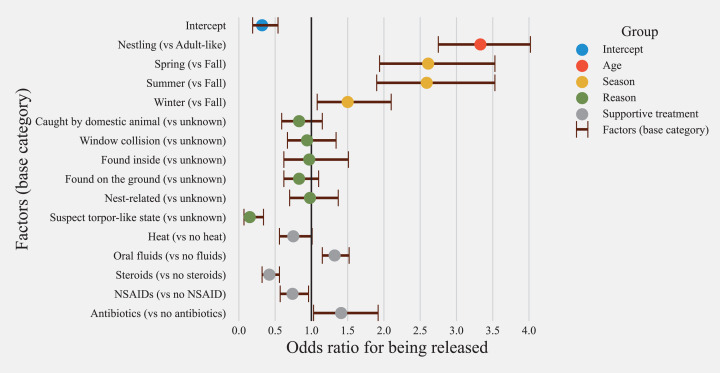
Risk factors and treatment options affecting the release of hummingbirds from rehabilitation centers. Odds ratios for all risk factors and their confidence intervals for the subset of hummingbirds with treatment information available in the database (rescued hummingbirds’ *n* = 3779). Categories are color-coded according to the group of independent variables (Heat = supportive care and/or shock treatment; Oral Fluids = hydration and/or energy supplementation; NSAID = Non-steroidal anti-inflammatory drug; Steroids = Anti-inflammatory and antipyretics).

**Figure 7:**
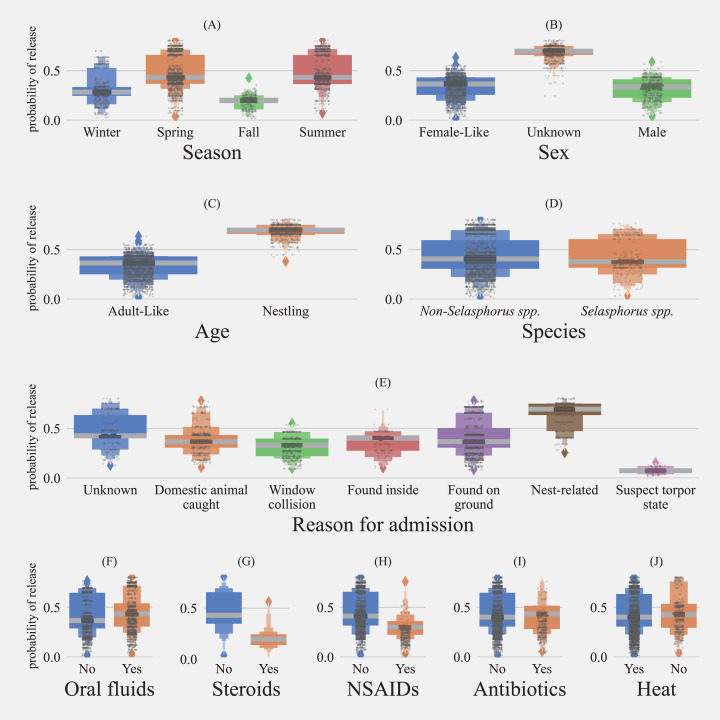
Predicted probabilitiy of release for hummingbirds. Distributions of predicted probability using the model vs risk factors for the subset of birds with available treatment information. Boxplots show the median, quartiles, and outliers (diamonds). Each gray dot represents the probability of survival of individual hummingbirds ((A) Season; (B) Sex; (C) Age; (D) Species; (E) Reasons for admissions; (F) Oral Fluids = hydration and/or energy supplementation; (G) Steroids = Anti-inflammatory and antipyretics; (H) NSAID = Non-steroidal anti-inflammatory drug; (I) Antibiotics; (J) Heat = supportive care and/or shock treatment).

Age was not a significant factor related to survival in the general model (Fig. 5). In the model evaluating treatment options (Fig. 6), nestlings had 3.43 higher odds of release (CI = 3.57–5.51, *p* > 0.001). Predicted probability of successful release for nestlings based on model with treatment options (model 2) was also significantly higher than adult-like birds (Nestlings: (mean = 0.69, SD ± 0.056, *n* = 1026), adult-like birds: (mean = 0.33, SD ± 0.178, *n* = 2753, Fig. 7). Hummingbirds rescued in spring, summer, or winter showed significantly higher odds of release compared with birds rescued in fall (Figs. 5 and 6). In the general model, birds rescued in spring were twice as likely to be released compared to birds rescued in fall (odds ratio: 2.73, CI [2.12–3.52], *p* < 0.001), while the model evaluating treatment options showed survival odds of 2.61 (CI [1.94–3.53], *p* < 0.001). Birds rescued in summer also showed similar odds to that of birds rescued in spring, and were twice more likely to be released than birds rescued in fall (model 1 = (odds ratio: 2.75, CI [2.11–3.57], *p* < 0.001), model 2 = (odds ratio: 2.59, CI [1.90–3.53], *p* < 0.001)). Birds rescued in winter also showed significantly higher odds of survival when compared with birds rescued in fall (model 1 = (odds ratio: 1.56, CI [1.17–2.09], *p* = 0.002), model 2 = [odds ratio: 1.50, CI [1.08–2.10], *p* = 0.016)). Season and age interaction terms included in model 1 showed statistically non-significant odds ratios (Fig. 5). Model-predicted probabilities (Fig. 7) for birds rescued in various seasons also showed similar trends, with higher probability of release for birds rescued in summer (mean = 0.49, SD ± 0.160, *n* = 1067) and spring (mean = 0.48, SD ± 0.17, *n* = 1763), and least probability of release for birds rescued in fall (mean = 0.19, SD ± 0.061, *n* = 333).

#### Reasons for admission

Hummingbirds that were rescued for reasons related to “torpor-like state” had significantly lower odds of release compared to hummingbirds for which reasons of rescue were unknown (model 1 = (odds ratio: 0.21, CI [0.11–0.38], *p* < 0.001), model 2 = (odds ratio: 0.15, CI [0.07–0.34], *p* <=0.001)). Similarly, within model 1, hummingbirds with a reason for admission related to domestic animals (odds ratio: 0.65, CI [0.53–0.79], *p* < 0.001), window hit (odds ratio: 0.65, CI [0.52–0.82], *p* < 0.001), and those which were found on the ground (odds ratio: 0.76, CI [0.65–0.89], *p* < 0.001) also showed significantly lower odds of release compared to hummingbirds with an unknown reason for admission (Fig. 5). “Nest-related” birds had the highest predicted probability for successful release compared to other admission reasons (mean = 0.65, SD ± 0.117, *n* = 575). 45.7% of total nestlings contrary to only 3.62 adult-like birds were admitted due to “nest-related” reasons. Since both the age and reason for admission affect survivability and are associated with each other, they are thought to act as confounders for each other. Hence, the inclusion of both variables in the model was warranted. The least probability of survival was found in birds rescued in “torpor-like state” (mean = 0.07, SD ± 0.032, *n* = 88) followed by “window collisions” (mean = 0.31, SD ± 0.103, *n* = 409). The model-predicted probabilities for release are presented in Fig. 7.

#### Supportive care and treatment

Model 1 indicated that provision of supportive treatment, in general, was not found to be significantly associated with improving the odds of successful hummingbird release (odds ratio: 0.90, CI [0.80–1.01], *p* = 0.064, Fig. 5), but when explored further by parsing out treatments into broad treatment options for select birds that received treatment, model 2 identified treatment options that increased the odds of successful hummingbird release (Fig. 6). Hummingbirds that received antibiotics were found to be one and a half times more likely to be released successfully (odds ratio: 1.41, CI [1.03–1.92], *p* = 0.031) compared to hummingbirds that did not receive antibiotics. Provision of oral fluids also increased the odds of successful release significantly (odds ratio: 1.32, CI [1.15–1.52], *p* < 0.001). Even after accounting for reasons for admission, hummingbirds that were administered steroids (odds ratio: 0.42, CI [0.32–0.56], *p* < 0.001) and NSAIDs (odds ratio: 0.74, CI [0.57–0.96], *p* = 0.021) were found to have reduced odds of survival.

## Discussion

This is the first study to evaluate the survival of hummingbirds, found in urban settings, undergoing rehabilitation and factors that affect survival for this popular group of backyard species and key pollinator group. We identified key anthropogenic threats and reasons that hummingbirds found in urban settings were to be admitted to wildlife rehabilitation centers. Reasons for admissions associated with anthropogenic pressures such as being associated with domestic animals, window collisions, and found inside human structures, which together constituted 25% of the total admissions were frequent but did not show any differences in influencing the odds of successful rehabilitation. The data also indicated that the provision of supportive treatments such as commercial nectar solution can significantly increase the odds of survival for rescued hummingbirds.

More than twelve percent cases were associated with domestic animals, of which almost all were reported to be caught by cats. Although it cannot be ascertained in our study that being caught by a cat was the primary reason these hummingbirds were admitted into the rehabilitation centers, as the birds could have been down for other reasons (e.g., hitting a window and falling on the patio to be eventually found by a house cat), it nevertheless cannot be denied that the large number of admissions under this category was concerning. Hunting attempts by cats are known to be lethal for birds (Dauphiné & Cooper, 2009) and have been known to cause conservation concerns in some hummingbird species in North America (Lepczyk, Mertig & Liu, 2004). Given this high mortality rate, public education on the importance of keeping cats indoor-only and reducing the population of free-roaming cats in the environment are both crucial for moving forward (Woods, McDonald & Harris, 2003). Belled collars (Ruxton, Thomas & Wright, 2002), constructing “catios” (Rudio & Fagan, 2015), using a collar-mounted electronic sonic device (Calver et al., 2007), or restricting the number of cats per household (van Heezik et al., 2010) have all been shown to significantly reduce the number of cat predations on birds. Campaigns such as the Humane Society of the United States’ “Cats Indoors! The Campaign for Safer Birds and Cats,” local “Trap, Neuter, Release” initiatives, and individual veterinary-client education about keeping owned cats indoors are efforts attempting to move this issue in the right direction and reducing impacts of cats on avifauna in urban habitats (Burton & Doblar, 2004; Schenk & Souza, 2014).

Windows and other glass panes around houses and buildings are another major cause of hummingbird injury that led to significantly decreased odds of their successful rehabilitation (odds ratio for rehabilitation 0.94, *p* > 0.05). The data showed an increased number of window collisions during early spring that can be associated with the territorial nature of breeding hummingbirds (Graham, 1997; Stiles, 1982) and the infusion of hummingbird gardens with more aggressive migratory hummingbird species (Dearborn, 1998; Klem, 1989). Hanging parachute cords (Klem & Saenger, 2013), netting or glass etching (Gelb & Delacretaz, 2006), use of non-reflective tinted glass (Evans Ogden, 1996), and placement of backyard feeders within one meter of windows (Klem, 1989; Klem & Saenger, 2013) are among various solutions that have been shown to be effective in reducing window collisions. Increasing canopy coverage and, shrub layers in suburban neighborhoods and urban parks, keeping migratory and other biological corridors free of potential hazards such as clear windows and electrical towers, and utilizing darker-pigmented window panes that are more easily avoidable by flighted birds are all ways that humans can do their part in reducing their overall negative impact on hummingbird populations (Schenk & Souza, 2014).

Many hummingbirds were also presented to the rehabilitation centers due to reasons related to the destruction of their nests, the third most common reason for presentation. In this study, the “nest-related” category included any nestling that was found on the ground for which repositioning in a nest was not possible, either due to the nest being too high in the tree or the rescuer unable to locate the nest. It also included nestlings that were found with the nest due to either wind blowing them off the tree or the tree was cut down and the nest was found. Lastly, it included nestlings that were brought into the rehabilitation center by observers who could watch the nest regularly (the nest being positioned on a tree right at the level of the window or being built on anthropogenic materials—Christmas lights and decorations, door handles, etc.), who realized they hadn’t seen the hen nearby for a “long time” and therefore assumed the nest had been abandoned. Therefore, “nest-related” hummingbirds, especially young ones, raise the question as to whether or not these birds truly needed human interventions, even though rehabilitators do an initial examination for the dependent nestling to check if the crop has nectar or diet items from the hen in the wildlife to determine if reuniting attempts should be made or not. Guidelines that were developed by a hummingbird wildlife rehabilitators for evaluating if a nestling has been abandoned should be consulted before intervention occurs (Greenway, 2020).

Hummingbirds found in a “torpor-like” state had the lowest probability of survival (odds ratio 0.45, *p* < 0.005). All records that mentioned the rescuer was able to capture the hummingbird without the bird attempting to escape were placed under this category. Hummingbirds can enter a state of torpor due to a variety of underlying reasons, such as physiological reasons, systemic illness, traumatic injury, severe dehydration, hypoglycemia, hypothermia, systemic shock, etc., or restricted energy resources (Hainsworth, Collins & Wolf, 1977). Entering torpor significantly slows the metabolism of these birds and, in turn, slows their ability to combat detrimental factors such as disease or injury (Hainsworth, Collins & Wolf, 1977). Given this, and the fact that it was an abnormal time of day for a hummingbird to be in torpor, we hypothesized that hummingbirds admitted in a suspected state of torpor do poorly due to their compromised overall condition.

The number of hummingbirds rescued showed seasonality, with a higher number of individuals presented at rehabilitation centers during the spring (odds ratio 2.76, *p* < 0.001) and summer (odds ratio 2.61, *p* < 0.001), correlating with the increased number of new nestlings (https://www.audubon.org/bird-guide) and first-year breeding individuals (Phillips, 1975) similar to trends observed in other bird species (Burton & Doblar, 2004; Heyden, 2005; Wimberger & Downs, 2010). We tested this association by the inclusion of an interaction term between the season and age variables which the models identified as non-significant but significantly improved the model fit predicting survival. The interaction term did not improve the model fit for the model that evaluated treatment (model 2). These results indicate that nestlings have higher odds of successful release than adult-like birds. Along with the increase in nestlings of breeding hummingbird populations, an increase in migratory hummingbird species also contributes to the seasonality of admission trends. Adult Black-chinned Hummingbirds migrate to California for breeding and producing offspring during spring and summer seasons, while Rufous Hummingbirds only migrate through California in spring heading north to their breeding grounds, and again in fall, heading south to their winter grounds in Mexico.

Initial clinical assessment of rescued hummingbirds by rehabilitation centers can be subjective and can change over the years. Hence, the clinical status of these birds cannot be ascertained solely on the reasons for admission. Still, our results indicated that some simple supportive measures, such as the provision of oral commercial nectar solutions, and as appropriate, antibiotics, improve survival odds of hummingbirds. The difficulty in providing medical care to small avian species, such as hummingbirds, lies in the fact that much is unknown regarding medication pharmacokinetics and pharmacodynamics and varying metabolic requirements for hummingbirds of varying stages of life (Bucher & Chappell, 1989).

Diseases, pesticide exposure/toxicosis (Baek et al., 2020; Bishop et al., 2018; Filigenzi et al., 2019; Godoy et al., 2013; Godoy, Tell & Ernest, 2014; Graves et al., 2019; Mikoni et al., 2017), and trauma by other wildlife species, and age all play a role in the overall success of treatment modalities. Those hummingbirds with more extensive injuries due to window collisions or being caught by a cat may be less likely to survive in general due to underlying external and internal trauma, particularly if damage to the skull or beak was sustained (Wimberger & Downs, 2010). However, we speculate that the lower survivability of birds receiving stabilizing treatments such as dexamethasone and NSAIDS in traumatic cases might be due to their debilitating physical state rather than drugs that were administered to them. This same thought process also applies to antibiotic administration given that treatment was most likely associated with some type of physical injury. Choice of treatment options is heavily dependent on clinical presentation, degree of physical injury, and shock, and it inherently affects survival chances of hummingbirds. Clinical data at a finer scale describing clinical presentation are needed to elaborate associations of drug choice and hummingbird release success.

Standardization of data collection methods, accurate identification of bird species, age and sex, and elimination of data entry biases that get combined by lack of categorization at data entry level are some of the limitations that, once fixed, could help to better understand the rehabilitation of hummingbirds found in urban habitats. Furthermore, identification of hummingbirds (appropriate speciation, gender, and age distribution) admitted to wildlife rehabilitation centers remains a challenge due to the complexity in the differentiation of species that are female-like in their appearance. Although overall treatment and rehabilitation protocols were not affected by hummingbird species or sex, unidentified categorization of individuals contributes to skewed demographics (age, sex, species). In addition, some of the categories such as “found on ground,” “torpor-like”-state, and “associated with domestic animal”, may have been a secondary rather than the primary cause.

A higher degree of ecological awareness and understanding of the impacts of urbanization on hummingbird populations are key to mitigate future anthropogenic threats (Mazaris et al., 2008). With increasing urbanization of wildlife habitats and human interactions with hummingbirds, analyzing rehabilitation trends of hummingbirds provides insights to better manage the rehabilitation of one of the most charismatic avian groups.

## Conclusion

Our results provide evidence of anthropogenic threats to hummingbirds in urban habitats. Anthropogenic reasons for hummingbird admissions such as physical injures due to domestic animals and collisions with windows constituted more than a quarter of cases reported by three Californian rehabilitation centers. We also highlight treatment options that can significantly help improve the rehabilitation success of hummingbirds and we also saw a clear indication that the provision of supportive treatments and medical aids such as the provision of commercial nectar solution can significantly increase the odds of survival of rescued hummingbirds.

## Supplemental Information

10.7717/peerj.11131/supp-1 Supplemental Information 1 Supplemental tables.

## Additional Information and Declarations

### Data Availability

The following information was supplied regarding data availability: The data and code are available at Zenodo: Pranav Pandit. (2020, December 8). PanditPranav/Hummingbird_Rehabilitation: PeerJ submission (Version v1.0). Zenodo. http://doi.org/10.5281/zenodo.4311820.
