# Supplementary material for: Retrospective study on admission trends of Californian hummingbirds found in urban habitats (1991–2016)
Source: PeerJ. 2021 Apr 13;9:e11131. doi: 10.7717/peerj.11131 (PMC8051342; doi:10.7717/peerj.11131)
Supplement: Supplemental Information 1 [file peerj-09-11131-s001.docx]

**Supplementary Table 1:** ANOVA test results for comparison of two model candidates (with and without season and age interaction term for Model 1.

|  | **Df** | **AIC** | **BIC** | **logLik** | **deviance** | **Chisq** | **Chi Df** | **Pr(>Chisq)** |
| --- | --- | --- | --- | --- | --- | --- | --- | --- |
| **Model 1 without interaction term*** | 14 | 8137.405 | 8233.171 | -4054.703 | 8109.405 | NA | NA | NA |
| **Model 1 with interaction term**** | 17 | 8131.188 | 8247.475 | -4048.594 | 8097.188 | 12.21737 | 3 | <0.001 |

***Model 1 without interaction term:** Disposition ~ Age + Season + Supportive Treatment + Reason for Admission + (1 | Species) + (1 | Sex)

****Model 1 with interaction term:** Disposition ~ Age + Season + Supportive Treatment + Reason for Admission + Age × season + (1 | Species) + (1 | Sex)

**Supplementary Table 2**: Summary table for mixed effect logistic regression model for predicting survivability of hummingbirds at rehabilitation centers with species and sex as random effect. Model evaluating the survivability of hummingbirds at rehabilitation centers in California, with model parameters indicating Odds ratio, 95% confidence interval and significance for all variables.

|  | **Model 1** | | |
| --- | --- | --- | --- |
| *Predictors* | *Odds Ratios* | *CI* | *p* |
| (Intercept) | 0.20 | 0.14 – 0.28 | **<0.001** |
| Age: Nestling | 0.85 | 0.10 – 7.13 | 0.877 |
| Season: Spring | 2.73 | 2.12 – 3.52 | **<0.001** |
| Season: Summer | 2.75 | 2.11 – 3.57 | **<0.001** |
| Season: Winter | 1.56 | 1.17 – 2.09 | **0.002** |
| Supportive treatment: Yes | 0.90 | 0.80 – 1.01 | 0.064 |
| Reason: caught by domestic animal | 0.65 | 0.53 – 0.79 | **<0.001** |
| Reason: window hit | 0.65 | 0.52 – 0.82 | **<0.001** |
| Reason: found inside | 0.86 | 0.62 – 1.19 | 0.351 |
| Reason: found on the ground | 0.76 | 0.65 – 0.89 | **0.001** |
| Reason: nest-related | 0.99 | 0.80 – 1.22 | 0.933 |
| Reason: suspect torpor-like state | 0.21 | 0.11 – 0.38 | **<0.001** |
| Age [Nestling] * Season [Spring] | 5.33 | 0.64 – 44.52 | 0.122 |
| Age [Nestling] * Season [Summer] | 3.91 | 0.47 – 32.77 | 0.208 |
| Age [Nestling] * Season [Winter] | 7.01 | 0.82 – 60.13 | 0.076 |
| **Random Effects** | | | |
| σ^2^ | 3.29 | | |
| τ_00_ _Species_ | 0.00 | | |
| τ_00_ _Sex_ | 0.01 | | |
| ICC | 0.00 | | |
| N _Species_ | 2 | | |
| N _Sex_ | 3 | | |
| Observations | 6908 | | |
| Marginal R^2^ / Conditional R^2^ | 0.179 / 0.182 | | |

**Supplementary Table 3:** ANOVA test results for comparison of two model candidates (with and without season and age interaction term for Model 2.

|  | **Df** | **AIC** | **BIC** | **logLik** | **deviance** | **Chisq** | **Chi Df** | **Pr(>Chisq)** |
| --- | --- | --- | --- | --- | --- | --- | --- | --- |
| **Model 2 without interaction term*** | 18 | 4649.608 | 4761.877 | -2306.804 | 4613.608 | NA | NA | NA |
| **Model 2 with interaction term**** | 21 | 4650.340 | 4781.321 | -2304.170 | 4608.340 | 5.267754 | 3 | 0.1532085 |

***Model 2 without inte**raction term: Disposition ~ Age + Season + Reason for Admission + Heat + Oral fluids + Steroid + NSAIDs + Antibiotic + (1 | Species) + (1 | Sex)

****Model 2 with interaction term:** Disposition ~ Age + Season + Reason for Admission + Heat + Oral fluids + Steroid + NSAIDs + Antibiotic + Age × season + (1 | Species) + (1 | Sex)

**Supplementary Table 4:** Summary table for best fitting mixed effect logistic regression model for predicting survivability of hummingbirds at rehabilitation centers with treatment options with species as random effect. The final model evaluating the survivability of hummingbirds at rehabilitation centers in California, with model parameters indicating Odds ratio 95% confidence interval and significance for all variables. Variables represent Sex, age category, season, categorical reason for admission, and various treatment options [Heat= supportive care and/or shock treatment; Oral fluids= hydration and/or energy supplementation; NSAIDs= Non-steroidal anti-inflammatory drug; Steroids = Anti-inflammatory and antipyretics].

|  | **Model 2** | | |
| --- | --- | --- | --- |
| *Predictors* | *Odds Ratios* | *CI* | *p* |
| (Intercept) | 0.32 | 0.19 – 0.54 | **<0.001** |
| Age: Nestling | 3.33 | 2.75 – 4.02 | **<0.001** |
| Season: Spring | 2.61 | 1.94 – 3.53 | **<0.001** |
| Season: Summer | 2.59 | 1.90 – 3.53 | **<0.001** |
| Season: Winter | 1.50 | 1.08 – 2.10 | **0.016** |
| Reason: caught by domestic animal | 0.83 | 0.59 – 1.15 | 0.259 |
| Reason: window hit | 0.94 | 0.67 – 1.34 | 0.748 |
| Reason: found inside | 0.97 | 0.62 – 1.51 | 0.876 |
| Reason: found on the ground | 0.83 | 0.62 – 1.10 | 0.189 |
| Reason: nest-related | 0.98 | 0.70 – 1.37 | 0.903 |
| Reason: suspect torpor-like state | 0.15 | 0.07 – 0.34 | **<0.001** |
| Heat | 0.75 | 0.56 – 1.01 | 0.058 |
| Oral fluids | 1.32 | 1.15 – 1.52 | **<0.001** |
| Steroid | 0.42 | 0.32 – 0.56 | **<0.001** |
| NSAIDs | 0.74 | 0.57 – 0.96 | **0.021** |
| Antibiotics | 1.41 | 1.03 – 1.92 | **0.031** |
| **Random Effects** | | | |
| σ^2^ | 3.29 | | |
| τ_00_ _G_O_ | 0.02 | | |
| τ_00_ _Sex_e_ | 0.00 | | |
| N _G_O_ | 2 | | |
| N _Sex_e_ | 3 | | |
| Observations | 3779 | | |
| Marginal R^2^ / Conditional R^2^ | 0.186 / NA | | |
